# Managing neurogenic orthostatic hypotension in a patient presenting with pure autonomic failure who later developed Parkinson disease

**DOI:** 10.1007/s10286-017-0428-4

**Published:** 2017-07-11

**Authors:** Fiona Gupta, Daniel Kremens, Steven Vernino, Beverly Karabin

**Affiliations:** 1 Movement Disorders Center, Hackensack University Medical Center, Hackensack, NJ USA; 2 Department of Neurology, Thomas Jefferson University, Philadelphia, PA USA; 3 Department of Neurology, UT Southwestern, Dallas, TX USA; 4 Syncope and Autonomic Disorders Center, University of Toledo, Toledo, OH USA

**Keywords:** Neurogenic orthostatic hypotension, Pure autonomic failure, Parkinson’s disease, Droxidopa

## Challenge questions

What are the criteria to begin pharmacologic therapy in patients with neurogenic orthostatic hypotension? What are the criteria to use one treatment or another?

## Case presentation

Mrs. A is a 50-year-old married woman who first presented 3 years ago for consultation because of recurrent orthostatic lightheadedness and two brief syncopal episodes. She reported lightheadedness within 5 min of standing, which resolved quickly on sitting or lying down. No history of cardiac disease was noted and her only medication at the time was hormone supplementation for menopausal symptoms. She denied symptoms of imbalance, tremor, constipation, or urinary dysfunction. On direct questioning of her spouse, a history was revealed of occasional shouting or kicking during the night, which they attribute to the “acting out” of vivid dreams. Vital signs revealed hypertension while supine and orthostatic hypotension (OH) without an appropriate compensatory increase in heart rate (HR). Supine blood pressure (BP) was 158/95 mmHg with an HR of 65 beats per minute (bpm). After standing for 3 min, the BP was 141/85 mmHg with an HR of 67 bpm. The remainder of her medical and neurologic examination was normal. Specifically, there were no features of parkinsonism, peripheral neuropathy, tremor, or cognitive impairment.

This patient presented with signs and symptoms suggesting sympathetic noradrenergic failure causing symptomatic neurogenic OH (nOH). However, she had no other features to suggest a diagnosis of Parkinson disease (PD), multiple system atrophy (MSA), dementia with Lewy bodies (DLB), or peripheral autonomic neuropathy. The presumptive diagnosis was pure autonomic failure (PAF, formerly known as Bradbury–Eggleston syndrome) [3]. Additional testing included electrocardiogram and echocardiogram. These tests ruled out cardiogenic causes of OH. Another useful test to help diagnose PAF is the measurement of supine and standing plasma norepinephrine levels. These are characteristically very low in patients with PAF and, most importantly, do not increase appropriately when standing, despite the severe fall in BP.

The patient was educated about nOH and non-pharmacologic measures were recommended, including fluid and salt loading, use of an abdominal binder, and elevating the head of the bed at night to help alleviate supine hypertension and also reduce nocturnal diuresis. These simple measures were sufficient to control this patient’s nOH for about a year.

Over time, patients initially thought to have PAF may develop features of parkinsonism and ultimately be diagnosed with PD, DLB, or MSA [3, 4]. Therefore, Mrs. A was advised to have periodic evaluations with her neurologist. Several years after initial presentation, the patient noted gradual worsening of balance and right hand tremor at rest. She also complained of worsening orthostatic symptoms. After a neurologic examination, Mrs. A was diagnosed with PD with OH.

She was prescribed rasagiline 1 mg and pramipexole dihydrochloride 1.5 mg daily, and she was instructed to continue the non-pharmacologic measures for her OH symptoms. At the patient’s most recent visit, she reported improvement in tremor, but marked fatigue as well as sleepiness, despite sleeping well at night. She also noted that she has had several falls but denies losing consciousness. Her motor function examination is unchanged from her prior visit. Her orthostatic supine-to-standing BP and HR were measured. After standing for 3 min, the systolic and diastolic BP dropped by 9 points (from 151/92 mmHg to 142/83 mmHg); she also showed a lack of adequate HR increase (71 bpm to 75 bpm) upon standing.

## Expert commentary (Dr. Vernino)

Although OH is often characterized by lightheadedness, dizziness, or syncope, it can often present with other non-specific symptoms such as generalized weakness, falls, leg buckling, fatigue, and neck pain, among other symptoms. These non-specific symptoms may be exacerbated by eating as a result of abnormalities in the splanchnic flow. Although the 9-point drop in systolic BP measured in the office does not reach diagnostic criteria for nOH, there should be a high index of suspicion in a PD patient previously diagnosed with nOH and other non-specific symptoms suggestive of nOH. Symptomatic nOH may not always correlate with isolated measurements of systolic BP because of the diurnal circadian rhythms of BP, as well as the fact that there are marked fluctuations in BP throughout the day in patients with nOH. In these cases, the use of a 24-h ambulatory blood pressure monitor is very useful [5].

## Expert commentary (Dr. Gupta)

This patient is experiencing symptoms that do not necessarily correlate with the classic motor symptoms of PD but are greatly troublesome. In this case, it is imperative to check for causes beyond the motor deficits of PD and, rightly so, orthostatic BP was measured. A 9-point drop in the office does not meet established criteria for OH, but the lack of adequate HR increase strongly suggests underlying nOH. I would encourage BP monitoring at home with seated or supine and standing measurements taken before arising in the morning and at bedtime. I believe the best outcomes with regards to the management of PD lie with multidisciplinary care; therefore, I would also contact the patient’s primary care physician and cardiologist to discuss my concerns.

## Case continuation

Medications, particularly dopamine agonists, can exacerbate nOH. In this patient, the dose of pramipexole dihydrochloride was lowered to 0.75 mg/day, but her symptoms did not improve. In addition, laboratory tests did not show evidence of chronic anemia, thyroid dysfunction, or hypovolemia.

The severity of OH varies during the day but may be most problematic in the morning. A single office measurement of orthostatic vital signs, particularly in the afternoon, may not reflect the severity of OH. A more effective evaluation of nOH would be to have the patient monitor their BP at home. Ambulatory 24-h BP monitoring can be very informative [5], although unfortunately this test is not widely available. For this patient, it was recommended that they take their own BP at home.

Mrs. A and her caregivers were educated regarding how to accurately measure and document BPs at home. It was recommended that the caregiver take the patient’s BP in a seated position (or supine if first thing in the morning prior to rising) and then again at 3 min after standing. They were asked to take the seated (supine in morning) and standing BPs and HRs in the morning (prior to rising, if possible) and then repeat seated and standing measurements 1 h after meals during the day and then again at bedtime.

Results of home BP monitoring showed significant OH at several times during the day without an adequate increase of HR. These measurements confirmed a diagnosis of nOH; therefore, the patient was advised that they no longer needed to take frequent BP measurements, but could do so if she was feeling symptomatic.

As troublesome symptoms of nOH persisted in this patient, the addition of pharmacologic therapy was warranted. Reasoning that PD and PAF patients with nOH have low plasma norepinephrine levels, droxidopa was considered an appropriate pharmacotherapy [1, 2]. Droxidopa treatment was initiated at 100 mg three times daily (TID) (prior to arising from bed, at lunchtime, and at mid-afternoon at least 3–4 h prior to bedtime). The dose was titrated to 400 mg TID.

After being on 400 mg droxidopa TID for 6 months, the patient reported a partial improvement in her symptoms of lightheadedness and weakness. She was then further titrated to 600 mg droxidopa TID, which resulted in further improvements in nOH symptoms. She also noted that she is no longer falling. She was able to resume activities such as walking and going out with friends.

## Expert commentary (Dr. Vernino)

Although BP measurements are useful to monitor for supine hypertension, the most important information to use when adjusting therapy is the severity of clinical orthostatic symptoms. The goal of therapy for nOH is not to normalize standing BP, but rather to relieve symptoms and improve quality of life.
